# 11β Hydroxylase Deficiency in a Child with Hypothyroidism: A Case Report

**DOI:** 10.31729/jnma.8378

**Published:** 2023-12-31

**Authors:** Bipesh Kumar Shah, Richa Koirala, Sumit Dhamala, Mukesh Bhatta, Shekhar Khatiwada

**Affiliations:** 1 Department of Paediatrics, B.P. Koirala Institute of Health Sciences, Dharan, Sunsari, Nepal; 2 B.P. Koirala Institute of Health Sciences, Dharan, Sunsari, Nepal

**Keywords:** *case reports*, *congenital adrenal hyperplasia*, *hypertension*, *hypothyroidism*

## Abstract

Congenital adrenal hyperplasia occurs due to enzymatic defects in the adrenocortical steroidogenesis. 11β hydroxylase deficiency is the second most common cause of congenital adrenal hyperplasia which presents with hypertension and features of androgen excess. Hypertension has also been found to cause end-organ damage in children with 11β hydroxylase deficiency. We report a case of a 10-year-old male child with hypothyroidism under thyroid replacement therapy, presenting with features of severe hypertension and androgen excess, later on, diagnosed as congenital adrenal hyperplasia due to 11β hydroxylase deficiency.

## INTRODUCTION

Congenital adrenal hyperplasia (CAH) results from defects in the adrenal steroidogenic pathway.^1^ 11β hydroxylase deficiency, with a prevalence of approximately 1 case per 100,000 live births, is the second most common cause of CAH.^2^ It accounts for 5 to 8% of cases.^3^ There exists a wide variability in the presentation of CAH.^4^ Depending on the percentage of enzyme activity and degree of severity, there exist two phenotypes, classic or non-classic, and the classic phenotype is associated with hypertension, virilization, and precocious pseudo-puberty.^5^ Hypothyroidism in children mostly occurs sporadically, however, dyshormonogenetic cases are recessively inherited.

## CASE REPORT

A 10-year-old male child, a known case of hypothyroidism under medication, thyroxine 50 mcg once daily, presented to the emergency department with severe hypertension; blood pressure (BP) of 160/120 mm Hg (>99^th^ percentile). Hypertension was never documented in the past. There was no history of hypertension, thyroid disorder, or renal disease in family members. His developmental history was comparable to that of his peer groups. Weight was 35 kg (75^th^-90^th^ centile), height was 127.5 cm (25^th^ centile), BMI was 21.53 kg/m^2^ (>27 adult equivalent) i.e., obese.

On examination, the child was conscious, and vitals apart from blood pressure were unremarkable. Head-to-toe examination revealed features suggestive of precocious puberty. The rest of the systemic examination was normal. The hypertensive emergency was managed initially with intravenous Labetalol and oral Clonidine. Thyroid function tests revealed slightly raised TSH (4.72 lU/ml), free T3 (3.69 pg/ml), and free T4 (17.1 pg/ml) were normal. Serum potassium (2.46) was decreased, while other electrolytes were normal in range. ECG was consistent with features of hypokalemia and left ventricular hypertrophy. Hypokalemia was managed with intravenous potassium chloride and potassium supplementation. On abdominal ultrasonography, bilateral kidneys showed increased cortical echogenicity. Urine examination revealed proteinuria of 1+. Renal artery stenosis was ruled out by renal artery Doppler. Echocardiography and fundus examination were normal. The dose of thyroxine was hiked to 62.5 mcg from 50 mcg. Both antihypertensives, Clonidine and Labetalol were tapered and stopped over a couple of days. Subsequently, Enalapril and Amlodipine, 5 mg each, once daily were added. The BP gradually maintained at 50th centile (100/70 mm Hg) over 2 weeks and the child continued with the antihypertensives.

Considering the features of hypertension, precocious puberty, and hypokalemia, the child was planned for steroid profiling and samples were sent for diagnosis. The reports revealed, increased levels of 11-Deoxycortisol (107 μg/L), 17-Hydroxyprogesterone (14.90 μg/L), 11-Deoxycorticosterone (13.70 μg/L), Progesterone (1.88 μg/L), 17-OHP + 21-Deoxycortisol: Cortisol ratio (2.47) and decreased level of cortisol (6.07 μg/L) which were suggestive of 11β hydroxylase deficiency. USG scrotum was also done which showed left testis enlargement with multiple heterogeneously hypoechoic lesions in bilateral testes with increased vascularity and tiny foci of calcification, likely to be testicular adrenal rest tumour. The child was started on hydrocortisone and has been doing well. The antihypertensives were stopped over a couple of months.

## DISCUSSION

According to a past study, 11β hydroxylase converts 11 deoxy-cortisol to cortisol and deoxy-corticosterone (DOC) to corticosterone and, in its deficiency, the decreased level of cortisol causes decreased feedback inhibition to the hypothalamus and anterior pituitary, increasing the secretion of adrenocorticotropic hormone (ACTH).^6^ This leads to increased levels of steroid precursors proximal to the enzymatic block; the resultant high levels of DOC cause sodium and fluid retention leading to hypertension.^5^ The elevated steroid precursors get directed towards androgen synthesis and subsequent androgen excess causes virilization and pseudo-precocious puberty.^7^

About two-thirds of patients with 11β hydroxylase deficiency present with hypertension and it is one of the differentiating features from 21-hydroxylase deficiency.^4^ Minority of patients have hypokalemia resulting from mineralocorticoid excess.^8^ End organ damage secondary to hypertension has been reported in patients with 11 hydroxylase deficiency CAH, including left ventricular hypertrophy, retinopathy, nephropathy, and cerebrovascular diseases.^9^ The renal damage seen in our case, can also be attributed to hypertension. Biochemical diagnosis of 11β hydroxylase deficiency includes elevated levels of DOC, 11-deoxycortisol, 17-hydroxyprogesterone, androstenedione, and testosterone with reduced levels of cortisol, renin, and aldosterone.^10^ Similarly, a past study stated that glucocorticoid, mostly in the form of hydrocortisone is the mainstay of therapy, which targets to replace the decreased cortisol and bring the level of ACTH to normal, thereby, preventing the overproduction of steroid precursors.^1^ The therapy aims to control blood pressure and prevent adverse effects of androgen excess.^1^ However, failure to control blood pressure despite optimal dosing of glucocorticoids, requires the addition of anti-hypertensive drugs.^5^ There exists significant challenges in the diagnosis of CAH due to 11β hydroxylase deficiency, considering its low prevalence and the lack of adequate laboratory facilities in a developing country like ours which adds to the financial burden of the patients making biochemical confirmation more challenging.

The diagnosis of CAH requires a high index of suspicion in young children presenting with features of androgen excess and hypertension. For a child with thyroid disorder presenting with severe hypertension and precocious puberty, other endocrine causes of hypertension like CAH should be ruled out apart from the renal causes.
